# Environmental suitability for *Aedes aegypti* and *Aedes albopictus* and the spatial distribution of major arboviral infections in Mexico

**DOI:** 10.1016/j.parepi.2019.e00116

**Published:** 2019-08-12

**Authors:** Jailos Lubinda, Jesús A. Treviño C., Mallory Rose Walsh, Adrian J. Moore, Ahmad Ali Hanafi-Bojd, Seval Akgun, Bingxin Zhao, Alassane S. Barro, Mst Marium Begum, Hera Jamal, Aracely Angulo-Molina, Ubydul Haque

**Affiliations:** a School of Geography and Environmental Sciences, Ulster University, Coleraine, United Kingdom; b Universidad Autónoma de Nuevo León, San Nicolás de los Garza, Nuevo Léon, Mexico; c Department of Public Health and Prevention Sciences, Baldwin Wallace University, Berea, OH 44017, USA; d Department of Medical Entomology and Vector Control, School of Public Health, Tehran University of Medical Sciences, Tehran, Iran; e Baskent University School of Medicine, Public Health Department, Baskent University, Turkey; f Department of Biostatistics, University of North Carolina at Chapel Hill, NC, USA; g African Group Organized for Research and Actions in Health, Burkina Faso; h Department of Pharmacy, East West University, Dhaka 1212, Bangladesh; i Department of Biology, University of Miami, Florida, USA; j Department of Chemical & Biological Sciences/DIFUS, University of Sonora (UNISON), Luis Encinas and Rosales S/N, Col. Centro, C.P. 83000, Hermosillo, Sonora, Mexico; k Department of Biostatistics and Epidemiology, University of North Texas Health Science Center, Fort Worth, TX 76107, USA

**Keywords:** Habitat suitability, MaxEnt, Niche modeling

## Abstract

**Background:**

This paper discusses a comparative geographic distribution of *Aedes aegypti* and *Aedes albopictus* mosquitoes in Mexico, using environmental suitability modeling and reported cases of arboviral infections.

**Methods:**

Using presence-only records, we modeled mosquito niches to show how much they influenced the distribution of *Ae. aegypti* and *Ae. albopictus* based on mosquito records collected at the municipality level. Mosquito surveillance data were used to create models regarding the predicted suitability of *Ae. albopictus* and *Ae. aegypti* mosquitos in Mexico.

**Results:**

*Ae. albopictus* had relatively a better predictive performance (area under the curve, AUC = 0.87) to selected bioclimatic variables compared to *Ae. aegypti* (AUC = 0.81). *Ae. aegypti* were more suitable for areas with minimum temperature of coldest month (Bio6, permutation importance 28.7%) −6 °C to 21.5 °C, cumulative winter growing degree days (GDD) between 40 and 500, and precipitation of wettest month (Bio13) >8.4 mm. Minimum temperature range of the coldest month (Bio6) was −6.6 °C to 20.5 °C, and average precipitation of the wettest month (Bio13) 8.9 mm ~ 600 mm were more suitable for the existence of *Ae. albopictus.* However, arboviral infections maps prepared from the 2012–2016 surveillance data showed cases were reported far beyond predicted municipalities.

**Conclusions:**

This study identified the urgent necessity to start surveillance in 925 additional municipalities that reported arbovirus infections but did not report *Aedes* mosquito.

## 1 Introduction

*Aedes* mosquitoes spread arboviruses (Hunter, 2016; WHO, 2014; WHO, 2017; Flores-Suarez et al., 2016), most of which have no known treatment. In the absence of therapeutic treatment or vaccination, prevention by targeting vectors is the optimal approach (WHO, 2017; von Seidlein et al., 2017; Reis et al., 2017). There is a higher risk and potential spread of viral infections, especially in urban areas, from *Aedes* mosquitoes. An increased probability of human exposure and habitat suitability support this proliferation of disease, which is often greater in more impoverished populations (WHO, 2017; Eisenstein, 2016; Cary Institute of Ecosystem Studies, 2017).

In Mexico, *Ae. aegypti* and *Ae. albopictus* are the primary mosquito species known for transmitting arboviruses (Flores-Suarez et al., 2016; Dzul-Manzanilla et al., 2016; Huerta et al., 2017) and have generally been reported ubiquitous (Ortega-Morales and Rodriguez, 2016; Kuri-Morales et al., 2017). They are both linked with the transmission of several arboviruses such as dengue, Zika, Chikungunya, and Yellow fever (von Seidlein et al., 2017; Benedict et al., 2007; Serpa et al., 2013).

Studies show that various methods of vector control have been implemented in Mexico since the 1950s (Flores et al., 2006), yielding mixed results over time (Flores-Suarez et al., 2016). These mixed results are mostly due to insecticide resistance to the chemicals used (Deming et al., 2016; Vazquez-Prokopec et al., 2017). However, vector control remains the most important and most promising method in reducing morbidity and mortality from arboviruses. Transmission methods of arboviruses are well known but local environmental factors driving the geographic distribution of *Aedes* species in Mexico have been explored but ignored the relationship with arboviral infections across mosquito-suitable areas; to date, those attempts to do so have focused primarily on the impact of elevation and temperature on *Ae. aegypti* (Lozano-Fuentes et al., 2012) or most recently *Ae. albopictus* (Yanez-Arenas et al., 2018) as single species at a time*.*

Among other arboviruses, dengue fever has recently been identified as the most prevalent (Mackenzie et al., 2004; Messina et al., 2014), causing disease in tropical and sub-tropical regions of the world, and expanding synchronously with the spread of *Aedes* vectors through human migration and trade in Asia, the Americas, Pacific and the Caribbean islands (Reis et al., 2017; Eisenstein, 2016; Calvez et al., 2016; Powell and Tabachnick, 2013). There is an increased risk of further potential expansion in Latin America in addition to a possible introduction in some European countries (Ibanez-Justicia et al., 2017; Kraemer et al., 2015a) where recent environmental suitability models have purported an increased likelihood of habitation by *Ae. aegypti* and *Ae. albopictus* due to warming temperatures because of climate change.

Although *Ae. aegypti* and *Ae. albopictus* are both primary arbovirus vectors (Powell and Tabachnick, 2013; Raharimalala et al., 2012), their ecological preference and survivorship differ significantly. The spatial distribution of *Ae. aegypti* is known to be confined within some temperate climates and most tropical, and subtropical climates, while the latter can also survive in areas with much cooler temperature ranges (Brady et al., 2013; Bonizzoni et al., 2013; Gardner et al., 2017). Literature shows that despite *Ae. aegypti*'s recognized restrictions based on precipitation and temperature (Lozano-Fuentes et al., 2012), more likely contributors to making this a primary vector (e.g., preference for humans, biting rate and gonotrophic discordance)*. Ae. aegypti'*s anthropophilic foraging preference exposes it to more human hosts than *Ae. albopictus,* which can feed on either humans or animals. However, in some parts of the world, studies have reported a distribution and invasiveness (Raharimalala et al., 2012; Juliano et al., 2004) of *Ae. albopictus* in relation to *Ae. aegypti*. It suggests that while the presence of either species is risky, the presence of both calls for much better surveillance mechanisms and a higher level of alarm.

With the potential risk of future climate change, the threat of the expansion of *Aedes* species may pose a formidable threat in their global geographic distribution and an increase in the various diseases they transmit. For this reason, environmental suitability modeling is crucial to understanding their current local distribution and to measuring the potential risk of *Aedes* being established beyond their current geographical domains. This study will also identify the gaps between current *Aedes* species and arbovirus surveillance. It will also identify the locations that need to bring urgently under surveillance in high disease burden and predicted risk areas where it might be informative about disease control program.

This study was designed to model environmental suitability in order to predict the potential geographic ranges for *Ae. aegypti* and *Ae. albopictus* in Mexico and compare with reported cases of chikungunya, dengue, and Zika virus infections from 2012 to 2016. The outcomes of this study will inform Mexican public health officials about areas suitable for the existence and establishment of the *Aedes* mosquito, including areas where they have not been previously reported, as well as providing information to support evidence-based strategies, interventions and vector surveillance plans for continued monitoring against outbreaks and potential spread into new areas.

## 2 Methodology

### 2.1 Mosquito presence data

Historical mosquito occurrence data records obtained from the Institute of Epidemiological Diagnosis and Reference (InDRE), were supplemented by those downloaded from the Dryad Digital Repository (Kraemer et al., 2015b). Presence points of *Ae. aegypti* and *Ae. albopictus* at the municipality level were compiled from 1993 to 2016 across all 2438 municipalities in Mexico. This dataset was collected and reported based on the Mexican national vector surveillance guidelines. The working definition of recorded ‘presence’ was determined by the availability of a report for at least one year between 1993 and 2016. During that period, records from 164 municipalities reported the presence of *Ae. albopictus* and 682 municipalities reported the presence of *Ae. aegypti*. The geographic information systems (GIS) package ArcGIS version 10.5, (Environmental Systems Resource Institute; [ESRI], Redlands, CA) were used to create municipality-based (for target intervention for each municipality) shapefile centroids in UTM projection system to which the recorded surveillance data was appended.

### 2.2 Arboviruses data

In this study, presence of infection for Chikungunya virus (CHIKV), Dengue virus (DENV), and Zika virus (ZIKV) was based on molecular methods used by the Mexican disease surveillance and notification system. For Chikungunya, infection in acute samples was determined using RT-PCR, and anti-CHIKV ELISA IgM. DENV confirmation was done either by virus isolation, viral RNA detection (PCR), NS1 antigen detection (ELISA), ELISA IgG, or by detection of IgM (ELISA). Finally, ZIKV test of positivity was done using RNA by PCR, using the QIAamp Viral RNA Mini Kit (QIAGEN, Hilden, Germany) (Diaz-Quionez et al., 2016). Real-time RT-PCR was also performed using the Superscript III system and viral NS5 gene coding (Jimenez Corona et al., 2016). All confirmed cases were reported within 24 h of detection at a local facility. Records were further communicated to the general directorate of epidemiology at the national level and processed by the National Reference Laboratory *(Institute for Epidemiological Diagnosis and Reference, InDRE)* and State Public Health Laboratories.

### 2.3 Source of the data

The main bioclimatic input variables (Source: Oak Ridge National Laboratory Distributed Active Archive Center, Oak Ridge, Tennessee, USA) in the models are listed in Table 1. The cumulative growing degree days (GDDs) during the period December–February was among the predictor variables retained in *Ae. aegypti* environmental suitability models.

**Table 1:** Predictor variables considered as candidate variables in *Ae. aegypti* and *Ae. albopictus* environmental suitability models.

Variable	Permutation importance (%)		
	Abbreviation	*Ae. aegypti*	*Ae. albopictus*
Temperature seasonality	BIO4	9.1	14
Maximum temperature of warmest month	BIO5	9.3	6.7
Minimum temperature of coldest month	BIO6	28.7	52.7
Precipitation of wettest month	BIO13	18.4	12.4
Precipitation seasonality (coefficient of variation)	BIO15	–	14.1
Cumulative GDDs during December-February	GDDs	34.5	–

Permutation importance values are given for variables selected in the final models.

Daily precipitation and temperature data from 1993 to 2016 was acquired from NASA with a spatial resolution of 1000 m × 1000 m (Thornton et al., n.d.) and compiled using Daymet ver.3 (Thornton et al., 2017) to calculate daily growing degree-days (GDDs). GDDs are an accurate thermal unit measure of the magnitude of daily temperatures accumulated above the 10 °C base temperature (Herms, 2004; Murray, 2008). GDDs of winter months (December–February) from 1993 to 2016 were derived by aggregating and averaging total winter warmth over the study period.

The mean monthly precipitation and temperature variables were calculated against the daily data dataset. Based on the derived mean monthly data, the nineteen most commonly used bioclimatic variables were calculated using the ‘dismo’ package in R version 1.1 (Hijmans et al., 2017). Temporal averages of climate variables were calculated using the Zonal Statistics tool in ArcGIS, 10.5 and overlaid against municipality-level ESRI's shapefiles. A matrix from Pearson's correlation coefficient was generated for each variable, and correlation strengths were tested before applying them using the ArcGIS statistical tool for multivariate band collection. The testing was used to help identify redundant [highly auto-correlated] (*r* > 0.80) variables so that they could be excluded from the final model analysis. All variables with correlation coefficients <0.8 and indicating apparent a prior relevance to *Aedes* biology was included in final model selection and analysis.

### 2.4 Normalized Difference Vegetation Index (NDVI), elevation, population density, and humidity data

Additional environmental and topographical variables (Humidity, NDVI, and Elevation) and social (population density) variables were added to improve explore the model. SPOT VGT Global NDVI dataset was downloaded from the Global Land Service on Copernicus website for the period 1998–2016. The downloaded data was in NetCDF format of 1 km spatial resolution and 10-day temporal resolution. We processed the data using free R programming (Team, 2013) extracting it by cell and aggregating it at municipality level. Elevation data at 1 km spatial resolution was extracted from SRTM imagery downloaded from USGS and extracted using ESRI ArcGIS 10.5 tools. Humidity data was derived from daily Daymet Vapor Pressure records and converted using Bolton's (Bolton, 1980) computation formula. Relative humidity was calculated from Vapor Pressure records as Relative Humidity = 100 * (vapor pressure / saturated vapor pressure). Finally, population density dataset was calculated based on the 2010 Mexico Population and Housing Census 2010 and estimated based on official projection estimates.

### 2.5 Modeling using MaxEnt

All suitability models were developed using the maximum entropy modeling approach with MaxEnt 3.4.1 (Phillips et al., n.d.). MaxEnt is a statistical modeling technique (Bellamy et al., 2013), using machine learning to estimate species ‘geographic distribution (Phillips, 2017). Climatic niches of *Aedes* species were defined by relating occurrence data to the set of input environmental predictors, and finding the distribution of *Aedes* occurrence probabilities of maximum entropy (closest to uniform); the computation procedure is constrained by the incomplete knowledge about the distribution of the *Aedes* species. The resulting niche models were then projected onto the geographic landscape of Mexico to predict habitat suitability for *Ae. aegypti* and *Ae. albopictus.* We chose MaxEnt over other softwares because it is well distinguished in species distribution studies (Dicko et al., 2014; Elith et al., 2011), and consistently out performs other reputable methods (Phillips et al., n.d.; Thibaud et al., 2014).

Preliminary results facilitated the exclusion of variables of permutation importance, and supported the preparation of key variables for each species. We set 1500 iterations, ran 10-fold cross-validation models of each species, and made sure one presence point at the municipality level was used in the model building and evaluation in order to ensure model convergence. Permutation importance captured high impact variables in each model, while jackknife plots were utilized to assess the predictive power of environmental variables. Jackknife and response curves further contribute towards our understanding of changes in the models after inclusion, exclusion or permutation of each variable. We used receiver operating characteristic curves (ROC) to measure variable-accuracy impact while the additional use of Jackknife plots determined which variables to contributed exclusive information.

For the overall model, fit assessment was derived from the area under the curve (AUC) of the ROC. The AUC range measured the probability that an occurrence point would rank above a randomly chosen background point (between 0.50 for random and closer to 1.0 for a better correlation) during multiple model performance comparisons. ROC was used to indicate true positive rates ‘*sensitivity*’ against the false positive rates ‘*specificity*’ as trade-offs in relation to model probability threshold values. We further applied ROC to measure the dualistic performance power of our model outcomes to show suitability or dearth.

Continuous surface maps using the municipality level point centroids were created to show suitability predictions for both species (e.g. the probability of *Aedes* species occurrence) using ArcGIS 10.5. Suitability maps were generated as a continuous dichotomy visualizing species distribution models with probability values close to 0 indicating areas unsuitable for the occurrence of the species, and higher values are associated with higher degree of habitat suitability for the species.

Finally, maps were created from reported chikungunya, dengue and Zika virus infections data to compare the presence of *Ae. aegypti* and *Ae. albopictus* mosquitos and predicted habitat suitability models.

## 3 Results

Out of 23 variables, six (Table 1) were found to be suitable using Pearson correlation (*r* < 0.8): temperature seasonality (Bio4), maximum temperature of warmest month (Bio5), minimum temperature of coldest month (Bio6), precipitation of wettest month (Bio13), precipitation seasonality (coefficient of variation) (Bio15), and cumulative growing degree days (GDDs) during December – February.

**Fig. 1:**
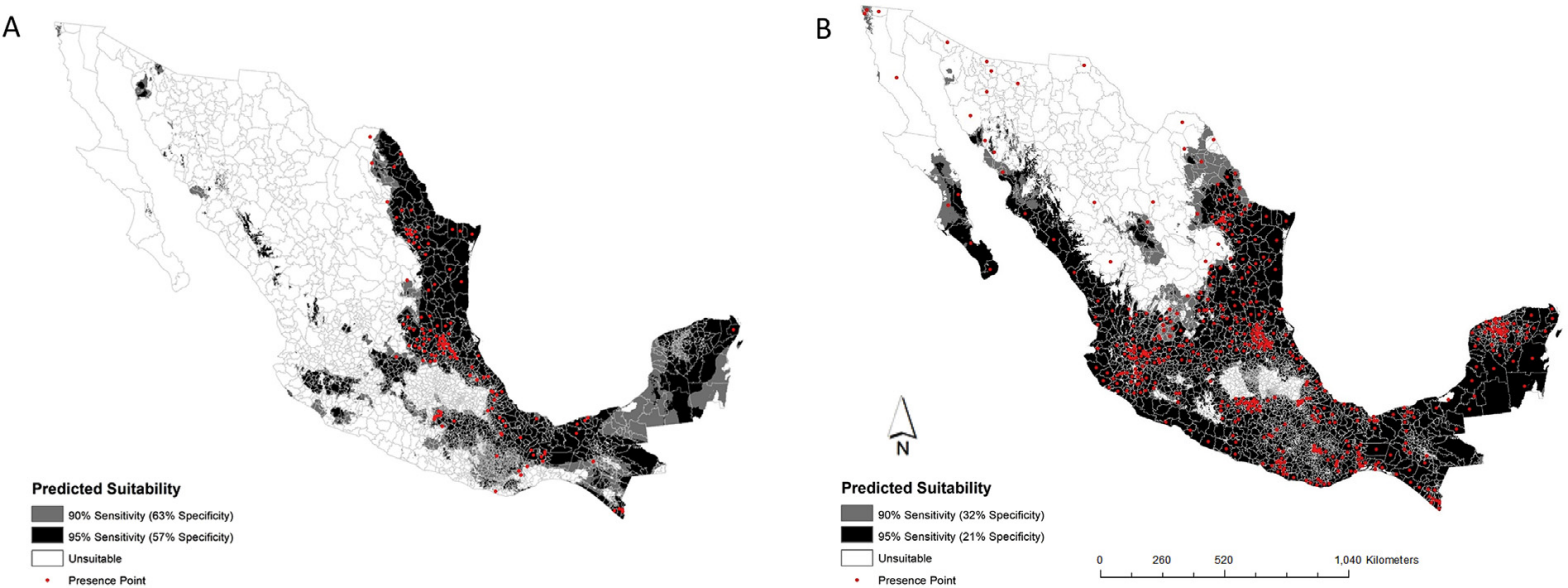
Showing probability maps of (a) *Ae. aegypti* and (b) *Ae. albopictus* at 90 and 95% sensitivity. Darker colour indicates a greater probability of suitability and lighter shading indicates less probability. Red points show the presence records from 1993 to 2016 used to build the models.

Results from this study with 90 or 95% prediction sensitivity, indicate significant overlaps in the modeled environmental suitability for both *Ae. aegypti* and *Ae. albopictus* in Mexico. *Ae albopictus* has a relatively smaller range of suitability extending from the southern-most tip of Mexico's north along the Gulf of Mexico to the US-Mexico border (Fig. 1). Although the whole region along the Gulf of Mexico indicates higher suitability (95% sensitivity) compared to the rest of the country, suitability sensitivity decreases to 90% from north to south-west with limited in-country suitability.

In comparison, and in addition to being suitable to the same areas as *Ae. aegypti*, *Ae. albopictus* has a much larger suitability range across the whole country covering more than double the area suitable for *Ae. albopictus,* including areas along the Pacific Ocean but most prominent in the southern half of the country (Fig. 1).

Table 1 shows the important variables selected for the final environmental suitability models. The selected models indicate that the three key variables of importance to the permutations (based on threshold values) for both species were: temperature seasonality (Bio4), minimum temperature of coldest month (Bio6), and precipitation of wettest month (Bio13).

Model results indicate that the mean AUC for *Ae. aegypti* was 0.81, with the variable of highest permutation importance (34.5%) being winter cumulative GDDs during December–February. However, in the *Ae. albopictus* model, this variable was of little permutation importance and was excluded. For *Ae. aegypti*, other variables with relatively high permutation significance included minimum temperature of coldest month (Bio6) and precipitation of wettest month (Bio13) with 28.7% and 18.4%, respectively (Table 1). Bio6 had a relatively higher permutation importance for *Ae. albopictus* (52.7%) with a mean AUC = 0.87. The variables Bio4, Bio5, and Bio15 (Table 1) were of comparatively low permutation importance for both species and show little differences between the species. Precipitation seasonality (coefficient of variation, Bio15) had the opposite effect having no significant permutation importance for *Ae. aegypti* but relatively higher for *Ae. albopictus*.

**Fig. 2:**
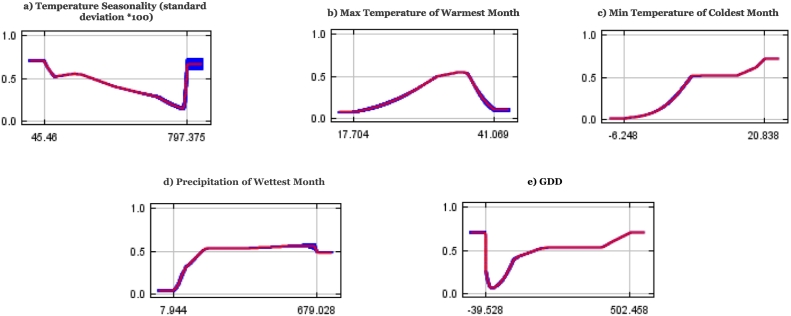
Model response curves for *Ae. aegypti* suitability in relation to (a) Temperature Seasonality (b) Maximum temperature during the warmest month (c) Min Temperature of Coldest Month (d) Precipitation of Wettest Month e) winter GDDs . Each curve shows the mean response of the ten replicate MaxEnt runs (black line) and the mean ± 1 standard deviation (dark shading) and represents how the logistic probability changes as each variable is changed while keeping all other variables at their average sample value.

Fig. 2(a–e) shows response curves of how each environmental variable affected the model prediction (Murray, 2008) by indicating how it changes as ecological-environmental variables are varied one after the other while keeping all other variables at their sample means.

In Fig. 2, model response curves for *Ae. aegypti* suitability for minimum temperature of coldest month [c], precipitation of wettest month [d], and winter GDDs [e] contributed significantly to the suitability model, exhibiting a curve closer to randomness. In short, an average minimum temperature of −6 °C to 21.5 °C during the coldest month, precipitation of wettest month >7.9 mm, and a winter cumulative GDDs (December–February) between 40 and 502 created the highest suitability for *Ae. aegypti*.

**Fig. 3:**
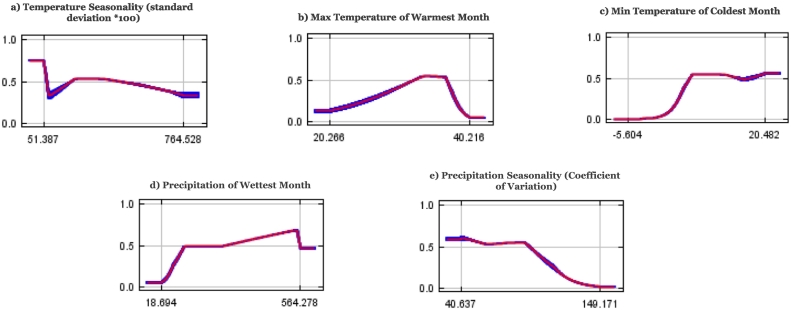
Model response curves for *Ae. albopictus* suitability in relation to (a) Temperature seasonality (b) Maximum temperature during the warmest month (c) Min temperature of coldest month (d) Precipitation of wettest month (e) Precipitation seasonality. Each curve shows the mean response of the ten replicate MaxEnt runs (black line) and the mean ± 1 standard deviation (dark shading) and represents how the logistic probability changes as each variable is changed while keeping all other variables at their average sample value.

Response curves for *Ae. albopictus* suitability (Fig. 3a–e) show that, maximum temperature during the warmest month [b], minimum temperature of coldest month [c], and precipitation of wettest month [d] had the most significant influence. The model revealed high suitability for areas with an average of ≥18.7 mm of rainfall during the wettest month. In summary, winters with minimum temperatures > − 5.6 °C, maximum temperature during the warmest month 20.3 °C – 40.2 °C, and with at least 18 mm of precipitation during the wettest month were significantly suitable for *Ae. albopictus.*

**Fig. 4:**
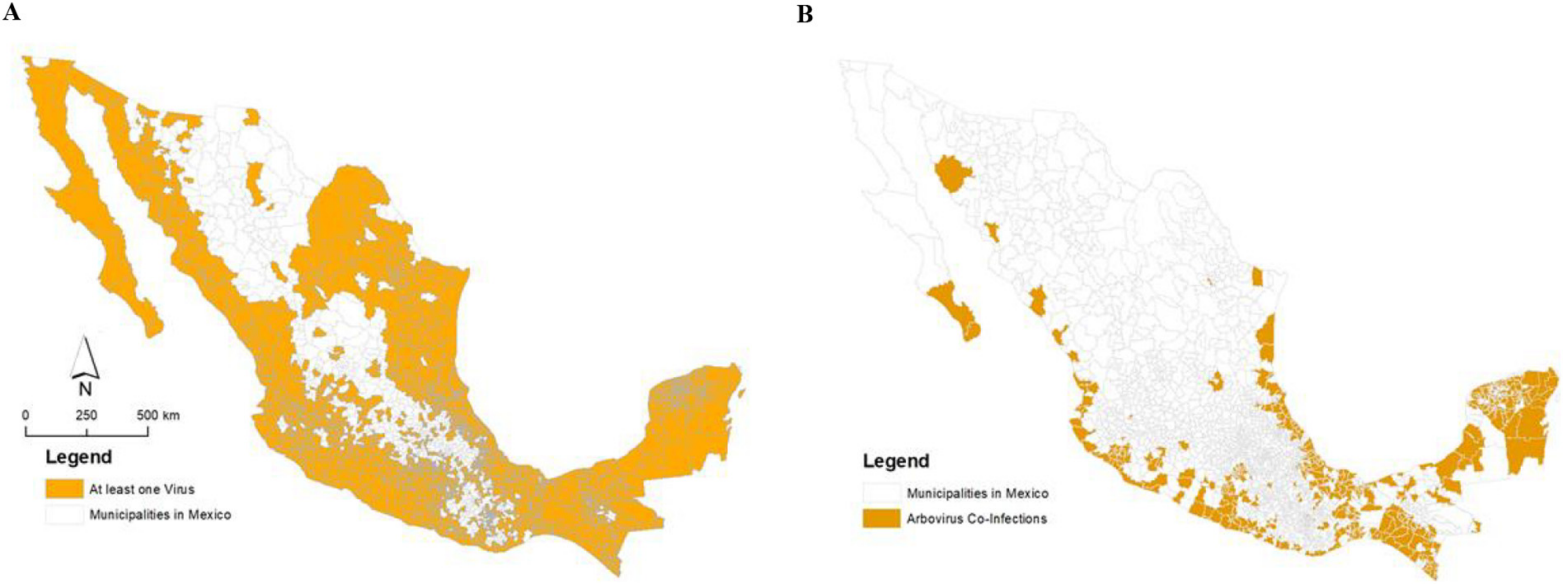
A. Municipalities (1437/2511 = 57%) reported at least one case of Chikungunya, Dengue, or Zika virus infection during 2012–2016 B. Municipalities (428/2511 = 17%) reported all three infections of Chikungunya, Dengue, and Zika virus during 2012–2016.

The maps (Fig. 4) indicate that arboviral infections reported (1437/2511 = 57% municipalities) are far beyond the municipalities reported both *Aedes* species (512/2511 = 20%) and predicted municipalities (Fig. 1). Based off of these results, it is evident that there is a wide gap between disease cases found and the predicted model regarding the presence of these mosquitos. This public health gap expresses a major concern for the future.

## 4 Discussion

In this study, we used Mexican national records of presence to explain the environmental suitability of *Ae. aegypti* and *Ae. albopictus*. The AUC for both species showed a positive suitability (probability >0.50) but *Ae. albopictus* had relatively higher sensitivity (AUC = 0.87) than *Ae. aegypti* (AUC = 0.81). For both species, the response curve suitability relative to the maximum temperature of the warmest month (Bio5), and a minimum temperature of the coldest month (Bio6) had comparable influence. Although cumulative winter GDDs was the highest suitability determinant for *Ae. aegypti,* it was not applicable to *Ae. albopictus,* and therefore did not make the final model selection.

Most parts of Mexico, especially the lower southern half of the country, have the highest probability of *Aedes* vectors surviving and reproducing during the warm period of the year. As such, public health decision makers can use the minimum precipitation of wettest month (Bio13) ≥ 8 mm to prepare for a potential increase in the probability of a community's suitability of either one or both *Aedes* species making imminent outbreaks of arboviruses. The identified areas must be considered as areas where the *Aedes* species are either present or could thrive and persist even though presence has yet to be reported.

Importantly, these species are thriving in areas in close proximity to human dwellings and breed in the water containers that people use for water storage (Thibaud et al., 2014; Getachew et al., 2015). Although the precipitation seasonality (coefficient of variation, Bio15) is of no permutation importance for *Ae. aegypti* but higher for *Ae. albopictus*, as observed earlier, it implies that *Ae. aegypti* has a more pronounced tendency towards human dwellings where it can oviposit in natural breeding sites than *Ae. albopictus*. Therefore, health education and campaign programs are highly recommended to encourage local communities to report mosquito larvae to the health authorities at the earliest possible time.

The essential parameters used in this study can be utilized as dynamic inputs into vector surveillance planning for target areas. Several studies have shown overlap of distribution of *Ae. aegypti* and *Ae. albopictus* (Kraemer et al., 2015a; Morrison et al., 2004), signifying different geographic and ecologic habitats, our study demonstrates substantial habitat overlap for these species. This suggests that despite the potential of competitive exclusion for hosts (interspecific competition) (Raharimalala et al., 2012; Bonizzoni et al., 2013; Juliano et al., 2004; Lounibos et al., 2010; O'Neal and Juliano, 2013; Yee et al., 2014; Chouin-Carneiro and Santos, 2017), huge habitat overlaps at both 90 and 95% sensitivity occur between *Ae. aegypti* and *Ae. albopictus* within given ecological thresholds (biotic and abiotic) as shown in our prediction maps (Fig. 1).

This suggests a more rigorous surveillance effort should be deployed primarily in regions of overlap for both species, especially given that the probability of transmission initiating from either or both vectors is higher. The distribution of suitability for both species corroborates some previous studies (Kraemer et al., 2015a; Messina et al., 2016; Johnson et al., 2017) whose findings showed high suitability in the southern areas of Mexico or appearing to share the same geographic or bio-ecological characteristics.

Our results confirm that winter GDDs are associated with occurrence of *Ae. aegypti*. This means that an area is predicted to be suitable for *Ae. aegypti,* there must be at least one day per winter (December–February) with temperature > 10 °C. Temperature changes associated with climate change are likely to vary substantially regionally in the form of an increase in the number of cumulative winter GDDs, minimum temperatures of the coldest month (Alto and Juliano, 2001). Also, climate change will involve other factors including altered precipitation patterns which are predicted to alter container mosquito population dynamics (Johnson et al., 2017; Alto and Juliano, 2001).

Maps created from disease data (Fig. 4) suggests there is a major public health gap. There are 925 municipalities that reported Arbovirus infections but did not report *Aedes* mosquito.. The predicted suitability indicated specific areas of Mexico with high environmental suitability for *Ae. agypti* and *Ae. albopictus* mosquitos. The reported data indicates that arboviral infections spread far beyond predicted municipalities (Fig. 1). As of 2016 when this data was last collected, the presence of *Ae. albopictus* was much lower than the presence of *Ae. aegypti* found in Mexico. However, this public health gap between the predicted suitability and real world map indicate a potential for the increase in the presence of *both species* in Mexico, which could lead to detrimental health effects for people in this region. It is also likely that the lack of alignment of arboviral infections, presence of both *Aedes* species, and habitat suitability could be lack of surveillance. Also, it is possible that people became infected in other mosquito suitable areas but were diagnosed in mosquito unsuitable areas. This major public health gap (arboviral infections and habitat suitability) was missing in Yanez-Arenas et al. (2018) and Kraemer et al. (2015a). All these models substantially underestimated the distribution of *Ae. aegypti* (and possibly *Ae. albopictus* too) and were heavily affected by biases in *Aedes* surveillance.

Arbovirus diseases are most sensitive to climate due to the environmental conditions (Messina et al., 2016) such as increased rainfall and shifting of seasonal temperatures, becoming more suitable for these mosquitos. These environmental changes, likely resulting from climate change, encourage the emergence or re-emergence of arboviruses (Johnson et al., 2017). It is imperative that we take steps to inhibit this emergence and re-emergence of arboviruses by increasing surveillance to predict future outbreaks, as well as continuing tracking of the presence of *Ae. aegypti* and *Ae. albopictus* mosquitos in Mexico.

These results are complimentary to those other studies where there was a positive relationship between suitability of *Ae. aegypti* and the average minimum temperature of coldest season, average precipitation of wettest month (Bio13), and the cumulative winter GDDs. However, this study also validated global models (Kraemer et al., 2015a) of arboviruses using local higher resolution data, and reported disease surveillance data making them more usable at a finer scale (targeted intervention for each municipality).

We propose that identifying vector suitability areas is just the first step towards parametric environmentally-focused breeding site control and preventive measures. Early stage vector larviciding guidance about potential breeding site elimination and distribution may depend heavily on such knowledge relating to areas of high suitability. This all lends itself to supporting the development of improved entomological indices that are integrated and tailored to a more unified vector surveillance strategy considering dengue and the recent Zika and Chikungunya outbreaks, all caused by the *Aedes* mosquitoes. Understanding of the probability of vector onset can only improve our ability to target and achieve efficient vector control preparedness mechanisms that are integrated with disease monitoring.

Among other limitations, the models in this study do not predict impending human-vector contact risk, which is a product of complex dynamics such as the entomological inoculation rate, transmission thresholds, man-biting ratios, and the daily survival rate of the vector. Besides, these models are not meant to quantify the amount or density of mosquito vectors present in the areas deemed suitable, and any inference thereof would have to be made with caution. Finally, it must be noted that these models are predicting the geographic distribution of species based on environmental or climatic conditions alone (Bellamy et al., 2013; Elith et al., 2006), meaning, they may not reflect the ongoing vector control and interventions present in the areas.

## 5 Conclusion

The increasing threat posed by *Aedes* mosquitoes calls for urgent action to regulate which vector control tools and approaches offer robust control and value. Selected surveillance strategies must be prioritized and integrated in order to respond properly and effectively in those areas identified as suitable. Until these *Aedes* species are controlled, the struggle against arboviruses is far from over. The longer it takes, the more complex the problem will become, posing an even greater threat to public health. Already, arboviruses have caught the attention of public health experts (Kraemer et al., 2015a; Messina et al., 2016) due to their globally increasing geographic extent and numbers of people affected. By utilizing high spatial resolution maps, our study offers an up-to-date estimate of the current potential geographic distribution of *Ae. aegypti* and *Ae. albopictus* and wider gap between disease cases and suitable municipalities in Mexico. This paper will specifically help local public health officials to prioritize surveillance for *Aedes* vector mosquitoes and arbovirus diseases, especially in areas with poor entomological surveillance and limited financial resources.

## Abbreviations

GDD: Growing Degree Days
InDRE: Instituto de Diagnóstico y Referencia Epidemiológicos
ESRI: Environmental Systems Resource Institute
CHIKV: Chikungunya virus
DENV: Dengue virus
ZIKV: Zika virus
NDVI: Normalized Difference Vegetation Index
SRTM: Shuttle Radar Topographic Mission
USGS: United States Geological Survey
ROC: Receiver Operating Curves
AUC: Area Under Curve

## Declaration of competing interest

All the authors declare that they have no competing interests.
